# *Gata3* ^ZsG^ and *Gata3* ^ZsG-fl^: Novel murine *Gata3* reporter alleles for identifying and studying Th2 cells and ILC2s *in vivo*

**DOI:** 10.3389/fimmu.2022.975958

**Published:** 2022-11-16

**Authors:** Rama K. Gurram, Danping Wei, Qiao Yu, Olena Kamenyeva, Hyunwoo Chung, Mingzhu Zheng, Matthew J. Butcher, Juraj Kabat, Chengyu Liu, Jaspal S. Khillan, Jinfang Zhu

**Affiliations:** ^1^ Molecular and Cellular Immunoregulation Section, Laboratory of Immune System Biology, National Institute of Allergy and Infectious Diseases, National Institutes of Health, Bethesda, MD, United States; ^2^ Department of Gerontology and Respirology, Xiangya Hospital, Central South University, Changsha, Hunan, China; ^3^ Research Technologies Branch, National Institute of Allergy and Infectious Diseases, National Institutes of Health, Bethesda, MD, United States; ^4^ Transgenic Core, National Heart, Lung, and Blood institutes, National Institutes of Health, Bethesda, MD, United States; ^5^ Mouse Genetics and Gene Modification Section, Comparative Medicine Branch, National Institute of Allergy and Infectious Diseases, National Institutes of Health, Bethesda, MD, United States

**Keywords:** GATA3, type 2 innate lymphoid cells, T helper cell 2, reporter mouse model, gene regulation, *in vivo*, allergic responses

## Abstract

T helper-2 (Th2) cells and type 2 innate lymphoid cells (ILC2s) play crucial roles during type 2 immune responses; the transcription factor GATA3 is essential for the differentiation and functions of these cell types. It has been demonstrated that GATA3 is critical for maintaining Th2 and ILC2 phenotype *in vitro*; GATA3 not only positively regulates type 2 lymphocyte-associated genes, it also negatively regulates many genes associated with other lineages. However, such functions cannot be easily verified *in vivo* because the expression of the markers for identifying Th2 and ILC2s depends on GATA3. Thus, whether Th2 cells and ILC2s disappear after *Gata3* deletion or these *Gata3*-deleted “Th2 cells” or “ILC2s” acquire an alternative lineage fate is unknown. In this study, we generated novel GATA3 reporter mouse strains carrying the *Gata3* ^ZsG^ or *Gata3* ^ZsG-fl^ allele. This was achieved by inserting a ZsGreen-T2A cassette at the translation initiation site of either the wild type *Gata3* allele or the modified *Gata3* allele which carries two *loxP* sites flanking the exon 4. ZsGreen faithfully reflected the endogenous GATA3 protein expression in Th2 cells and ILC2s both *in vitro* and *in vivo*. These reporter mice also allowed us to visualize Th2 cells and ILC2s *in vivo*. An inducible *Gata3* deletion system was created by crossing *Gata3* ^ZsG-fl/fl^ mice with a tamoxifen-inducible Cre. Continuous expression of ZsGreen even after the *Gata3* exon 4 deletion was noted, which allows us to isolate and monitor GATA3-deficient “Th2” cells and “ILC2s” during *in vivo* immune responses. Our results not only indicated that functional GATA3 is dispensable for regulating its own expression in mature type 2 lymphocytes, but also revealed that GATA3-deficient “ILC2s” might be much more stable *in vivo* than *in vitro*. Overall, the generation of these novel GATA3 reporters will provide valuable research tools to the scientific community in investigating type 2 immune responses *in vivo*.

## Introduction

GATA3 is a critical transcription factor for the development of CD4 T cells and non-lymphoid tissue inducer (non-LTi) innate lymphoid cells (1, 2). Also, group 2 innate lymphoid cells (ILC2s) and type 2 T helper (Th2) cells rely on GATA3 expression for their fate determination and functions (3, 4). Both Th2 cells and ILC2s express high levels of GATA3 (5). Furthermore, GATA3 expression positively regulates the expression of several type 2 effector cytokines in both Th2 cells and ILC2s (2). Although ILC2s and Th2 cells secret similar effector cytokines and other regulatory molecules, signals that induce their development and activation are quite different (6). Nevertheless, ILC2s and Th2 crosstalk with each other during type 2 immune responses, including host defense against nematode infection and allergic inflammatory responses (7). A complete understanding of similar and differential functions of GATA3 in ILC2s and Th2 cells is important for gaining deep insights into type 2 immune responses.

The mouse *Gata3* gene consists of six exons located on chromosome 2. We have previously generated a conditional *Gata3* allele (*Gata3* ^fl^) by inserting *loxP* sequences on both ends of the exon 4 which encodes functional zinc finger domains (8). Using tamoxifen-inducible deletion of *Gata3* gene under *in vitro* conditions, we have reported that many type 2 related genes (such as *Il5, Il13, Areg*, and *Il1rl1*) are positively regulated by GATA3 in both ILC2s and Th2 cells in an *in vitro* culture system (2). Since many known markers for identifying ILC2s or Th2 cells are regulated by GATA3, it is difficult to conclude whether “ILC2s” and/or “Th2” cells die after GATA3 deletion *in vivo* or they may adopt an alternative phenotype. For this reason, how GATA3 controls gene transcription in ILC2s and Th2 cells *in vivo* cannot be easily studied.

Mature Th2 cells express high levels of T1/ST2 (IL-33 receptor α chain) on their surface (9), which allows Th2 cells to respond to IL-33 to produce type 2 cytokines independent of T cell receptor-mediated signaling (10, 11). T1/ST2 has been widely used as a cell surface marker for isolating Th2 cells from non-Th2 cell populations that are generated *in vivo* in mice. ILC2s preferentially express several cell surface markers, including T1/ST2, CD25, ICOS, and KLRG1 (3, 12–14). While T1/ST2 is a reliable marker for identifying and isolating both Th2 cells and lung ILC2s, its expression is completely dependent on GATA3 expression (2). Because of this, whether GATA3-deficient “ILC2s” and “Th2” cells can persist *in vivo* is unknown.

It has been shown that GATA3 may self-regulate its own expression during Th2 cell differentiation in an overexpression study (15). However, it is also reported that *Gata3* transcription does not depend on functional GATA3 under a Th2 environment (16). Thus, we speculate that a reporter under the control of the *Gata3* gene could be a useful tool for identifying ILC2s and Th2 cells *in vivo* after *Gata3* deletion. Although several GATA3 reporter systems are currently available (17–19), none of them offers the opportunity to study conditional *Gata3* deletion *in vivo*.

In this study, we generated a novel GATA3 reporter system in combination with the conditional *Gata3* allele (*Gata3* ^ZsG-fl^). The insertion of the ZsGreen reporter at the translation initiation site of GATA3 faithfully reflected the expression of endogenous GATA3. The two *loxP* sites flanking exon 4 encoding functional zinc finger domains allow us to delete the *Gata3* gene by an inducible Cre. This novel mouse strain allowed us to identify, monitor and isolate Th2 cells and ILC2s after an acute GATA3 deletion in these cells *in vivo*. Through the experiments using these mice, we found that GATA3 is not required to regulate its own expression in mature type 2 lymphocytes and that GATA3-deficient “ILC2s”, defined by high levels of ZsGreen expression, might be relatively stable *in vivo*.

Another GATA3 reporter mouse strain carrying the *Gata3* ^ZsG^ allele, in which ZsGreen insertion took place on the wild type *Gata3* allele, can be used for general purposes in identifying, visualizing, and isolating Th2 cells and ILC2s. The ZsGeen insertion in both reporter systems does not affect the expression of endogenous GATA3 or impair the development of ILC2s and differentiation of Th2 cells. Therefore, our new GATA3 reporter mouse strains carrying the *Gata3* ^ZsG^ or *Gata3* ^ZsG-fl^ allele are valuable tools for studying Th2 cells and ILC2s during type 2 immune responses *in vivo*.

## Material and methods

### Mice

All mice were 8-12 weeks of age when they were used under a protocol (LISB-8E) approved by the National Institute of Allergy and Infectious Diseases (NIAID) Animal Care and Use Committee. *Gata3* ^fl/fl^ mice (line 355) and *Gata3* ^fl/fl^-CreERT2 (line 8445) were obtained from the NIAID-Taconic repository. *Gata3* ^ZsG/+^ mice were generated on C57BL/6 background by the Mouse Genetics and Gene Modification section, NIAID. *Gata3* ^ZsG-fl/fl^ mice were generated on the *Gata3* ^fl/fl^ mice background by the Transgenic Core Facility, NHLBI. The details of the generation of these novel reporter strains are described below. Mice carrying the *Gata3* ^ZsG-fl^ allele were also bred to the *Gata3* ^fl/fl^-CreERT2 mice to generate the *Gata3* ^ZsG-fl/fl^-CreERT2 strain. *Gata3* ^ZsG/+^ mice were bred to *Cd4* ^Cre^ *R26* ^tdTomato^ to generate *Gata3* ^ZsG/+^ *Cd4* ^Cre^ *R26* ^tdTomato^ mice. Mice were bred and/or maintained in the NIAID specific pathogen-free animal facilities.

### BAC recombineering for *Gata3*-*ZsGreen-T2A* cassette insertion

The initial insertion of the *ZsGreen-T2A* gene cassette into the *Gata3* gene on a BAC was accomplished *via* recombineering. *galK* positive and counterselection strategy was used to achieve *ZsGreen-T2A* insertion at the translation initiation site of the *Gata3* gene. SW102 strain containing a BAC with the *Gata3* gene was used for all BAC recombineering steps, and the detailed methodology for this process has been previously described (20). The gene *galK* encodes the enzyme galactokinase, which plays a key role in metabolizing galactose as a carbon source. The following *galK* primers (indicated in lowercase) with 50 bp *Gata3* gene homology arms were used for amplifying *galK* gene.

*Gata3-galK* 5’F: CTCCCTACCCGCGAGGGTTCCGGGCCGGGCGAGAGGGCGCGAGCACAGGCGACGACcctgttgacaattaatcatcggca

*Gata3-galK* 3’R:

CGCGGGGTGATGGTGGCTCACCCAGCGCGGCTGGTCCGCAGTCACCTCCATtcagcactgtcctgctcctt

The resulting PCR product was introduced into SW102 competent cells by electroporation and the *galK* cassette was then inserted into the *Gata3* gene-containing BAC by homologues recombination. The *galK* cassette insertion is critical for the positive selection of bacteria on minimal media containing galactose as the only carbon source. The *galK* insertion at the translation initiation site of *Gata3* gene enables them to utilize galactose. Conversely, the non-transformed bacterial cells are eliminated due to the lack of ability to use galactose as a carbon source. This step is crucial for the positive selection of bacteria which are incorporated with *galK* insertion at the region of interest.

The following set of ZsGreen-T2A primers (indicated in lower case) with 50 bp length *Gata3* gene homology arm was used for amplifying the *ZsGreen-T2A* gene.

*Gata3-Zsgreen-T2A* 5’F:

CTCCCTACCCGCGAGGGTTCCGGGCCGGGCGAGAGGGCGCGAGCACAGGCGACGACatggcccagtccaagcac

*Gata3-Zsgreen-T2A* 3’R:

CGCGGGGTGATGGTGGCTCACCCAGCGCGGCTGGTCCGCAGTCACCTCCATtgggccaggattctcctc

The amplified cassette was purified and introduced into the bacterial cells by electroporation. During this step, the *galK* gene in the BAC was replaced by Zsgreen-T2A cassette by homologues recombination. The bacteria containing *Gata3-ZsGreen-T2A* BAC was then counter-selected on a media containing 2-deoxy-galactose. During this process, bacterial cells still expressing *galK* are eliminated due to the toxic substance generated by galactose kinase metabolizing 2-deoxy-galactose. This leads to the selection of bacteria with the *Gata3-ZsGreen-T2A* BAC.

### Generation of the *Gata3* ^ZsG/+^ and *Gata3* ^ZsG-fl/fl^ mouse strains

The *ZsGreen-T2A* sequence was introduced into the endogenous *Gata3* locus in mice using CRISPR/Cas9 technology (21). Briefly, a sgRNA (GAGCACAGCCGAGGACATGG), which cuts near the *Gata3* translation initiation codon (ATG), was made using ThermoFisher’s custom *in vitro* transcription service. The sgRNA (10 ng/ul) and donor *Gata3-ZsGreen-T2A* BAC DNA (5 ng/ul) were co-microinjected with Cas9 mRNA (20 ng/ul, purchased from Trilink Biotechnologies) into the pronuclei of zygotes collected from the *Gata3* ^fl/fl^ mice (line 355) (8). Injected embryos were cultured in M16 medium (Millipore-Sigma) overnight in a 37°C incubator with 6% CO_2_. The following day, embryos that reached 2-cell stage of development were implanted into the oviducts of pseudo-pregnant surrogate mothers. Similarly, the same sgRNA (10 ng/μl), Cas9 mRNA (20 ng/μl) along with BAC clone (2 ng/μl) were injected into the pronuclei of normal C57BL/6 females from Taconic. The embryos were cultured overnight in KSOM medium to two cell stage and then transferred into the CD1 pseudo-pregnant females. Offspring born to the foster mothers were genotyped by PCR and Sanger Sequencing. The expression of ZsGreen in CD4 T cells was confirmed by FACS analysis. Following the establishment of new reporter lines, the *Gata3* ^ZsG/+^ mice (on the WT C57BL/6 background) or the *Gata3* ^ZsG-fl/fl^ mice (on the C57BL/6 background but also carrying *Gata3* floxed allele) were maintained as hemizygous through crossing with wild type C57BL/6 or *Gata3* ^fl/fl^ mice.

### Genotyping by PCR

The *ZsGreen-T2*A knock-in allele in the offspring mice was screened by standard PCR. Briefly, the pinna samples from weaned mice were collected and digested with 100 μl of proteinase K digestion buffer (100 mM Tris-HCl, pH 8.5, 5 mM EDTA, 0.2% SDS, 200 mM NaCl, and 1 mg/ml of proteinase K) at 55 °C overnight. The reaction was then diluted with distilled water in 1:10, and 1 μl was used as a template for PCR. The following set of primers was used to distinguish wild-type allele from knock-in allele. 5’-TAGTCAGTCCTGGGCTCCTG-3’ (WT F), 5’-TCCAGCACAAGCTGACCC-3’ (ZsGreenF), 5’-CAGCGGATACTGAGCTTCCATGT-3 (Common R). The annealing step in the PCR was conducted at 57 °C. After the completion of successful PCR, the amplified product for the knock-in gene allele appears at around 284 bp, and the wild-type allele appears at 700 bp.

### Quantitative PCR for *Gata3* and ZsGreen mRNA

Total RNAs were isolated using RNeasy kit (Cat# 74104, QIAGEN). cDNAs from isolated RNAs were prepared with the QuantiTect Reverse Transcription Kit (Cat# 205311, QIAGEN). Quantitative PCR was performed on QuantStudio™ 7, Applied Biosystems by using FastStart Universal SYBR Green Master mix (Rox) (Cat# 4913850001, Roche). The following set of primers are used for quantifying *Gata3* and ZsGreen mRNA. 5’-CTCGGCCATTCGTACATGGAA-3’ (*Gata3*-exon 2 F), 5’-GGATACCTCTGCACCGTAGC-3’ (*Gata3*-exon 2 R), 5’-AAGGCAGGGAGTGTGTGAAC-3’ (*Gata3*-exon 4 F), 5’-TCGCTTGGGCTTGATAAGGG-3’ (*Gata3*-exon 4 R), 5’-CCCTTATCAAGCCCAAGCGA-3’ (*Gata3*-exon 4-5 F), 5’- CCCATTAGCGTTCCTCCTCC-3’ (*Gata3*-exon 4-5 R), 5’-AAGGCATCCAGACCCGAAAC-3’ (*Gata3*-exon 6 F), 5’-GGAGAGATGTGGCTCAGGGA-3’ (*Gata3*-exon 6 R), 5’-ATCTGCAACGCCGACATCAC-3’ (ZsGreen F), 5’-TGATCTTCTCGCAGGAGGGC-3’ (ZsGreen R).

### *In vitro* polarization of T helper subsets

Naïve CD4 T cells from pooled lymph node cell suspension were purified using the naïve CD4 T cell isolation kit (Miltenyi Biotec, 130-104-453) according to the manufacturer’s instruction. All the T helper subset polarization experiments were performed on 24-well plates. Plate-bound anti-CD3 (1 μg/ml) and anti-CD28 (3 μg/ml) was used to polarize cells under Th1, Th17, and Treg conditions. For Th2 polarization conditions, the Dynabeads™ Mouse T-Activator anti-CD3/CD28 (Theromofisher, 11456D) microbeads were used due to an inefficient differentiation of Th2 cells using the plate-bound system. Additional cytokines and antibodies were supplied with the complete RPMI 1640 media under different polarization conditions: for Th1, 10 ng/ml IL-12, 10 μg/ml α-IL-4, and 100 U/ml IL-2; for Th2, 10 ng/ml IL-4, 10 μg/ml α-IFN-γ, and 100 U/ml IL-2; for Th17, 2 ng/ml TGF-β, 20 ng/ml IL-6, 10 ng/ml IL-1β, 10 μg/ml α-IFN-γ, and 10 μg/ml α-IL-4; for Treg, 5 ng/ml TGF-β, 10 μg/ml α-IFN-γ, 10 μg/ml α-IL-4, and 100 U/ml IL-2. All cultures were incubated at 37°C in 5% CO_2_. After 3-4 days of polarization, cells were washed and placed in a new culture plate with complete RPMI 1640 media with 100 U/ml IL-2.

### Papain-induced airway inflammation

Protease papain is well known to induce airway inflammation through activating type 2 immune responses (22). Mice were briefly anesthetized with isoflurane followed by papain intranasal (*i.n.*) challenges (40 µg in 20 µl of PBS) on days 0, 1, and 2. Mice were then rested for 10 days and challenged again with papain on days 13, 14, and 15. Mice were analyzed with the BALF and lung tissues 24 hr after the final *i.n.* challenge.

### Flow cytometry analysis

*In vitro* cultured cells or cells isolated from the BALF and lung tissues were stained with different antibody cocktails for identifying eosinophils, Th2 cells, and ILC2s, and analyzed by FACS LSR-II or Fortessa (BD Bioscience). Foxp3/transcription factor staining buffer set (eBioscience) was used for identifying intracellular markers. Eosinophils were identified as CD45^+^CD11c^-^Gr1^-^SiglecF^+^CD11b^+^; Th2 cells were identified as CD4^+^Foxp3^-^GATA3^+^T1/ST2^+^; ILC2s were identified as Lin^-^IL-7Rα^+^GATA3^+^T1/ST2^+^. All flow cytometric data analysis was performed with FlowJo software.

### Live confocal microscopy of lung sections

Live confocal microscopy of lung sections was used for visualizing the distribution of ILC2s and Th2 cells in mouse lung tissue slices *ex vivo*. After euthanasia, mouse lungs were inflated with 1.5% of low-melt agarose in RPMI at 37°C. Inflated tissues were kept on ice, in 1% FBS in PBS, and sliced into 300-350 µm sections using Leica VT1000 S Vibrating Blade Microtome (Leica Microsystems) at speed 5, in ice-cold PBS. Tissue sections were stained with fluorescence labeled anti-EPCAM antibody (eBioscience) for 2 h on ice. After staining, sections were washed three times and cultured in complete lymphocyte medium (Phenol Red-free RPMI supplemented with 10% FBS, 25 mM HEPES, 50 μM β-ME, 1% Pen/Strep/L-Glu and 1% Sodium Pyruvate) in a humidified incubator at 37°C. Tissues were allowed to completely recover for 12 h prior to imaging. Sections were held down with tissue anchors (Warner Instruments) in 14 mm microwell dishes (MatTek) and imaged using Leica DMi8 inverted 5 channel confocal microscope equipped with an Environmental Chamber (NIH Division of Scientific Equipment and Instrumentation Services) to maintain 37°C and 5% CO_2_. Microscope configuration was set up for four-dimensional analysis (x,y,z,t) of cell segregation and migration within tissue sections. Diode laser for 405 nm excitation; Argon laser for 488 and 514 nm excitation, DPSS laser for 561; and HeNe lasers for 594 and 633 nm excitation wavelengths were tuned to minimal power (between 1 and 5%). Z stacks of images were collected (10 – 50 µm). Mosaic images of lung sections were generated by acquiring multiple Z stacks using a motorized stage to cover the whole section area and assembled into a tiled image using LAS X (Leica Microsystems) software. Post-acquisition mages were processed using Imaris (Bitplane) software.

### Statistical analysis

Data analysis was performed with GraphPad Prism (GraphPad Software). The student’s t-test was used to determine the statistical significance between the two groups. Data were presented as mean ± SEM. A p-value <0.05 was considered statistically significant and indicated as *; p < 0.01 was indicated as **; p < 0.001 was indicated as ***; and p < 0.0001 was indicated as ****. Not statistically significant was indicated as ns.

## Results

### Generation of novel *Gata3*-*ZsGreen* reporter mouse strains

**Figure 1:**
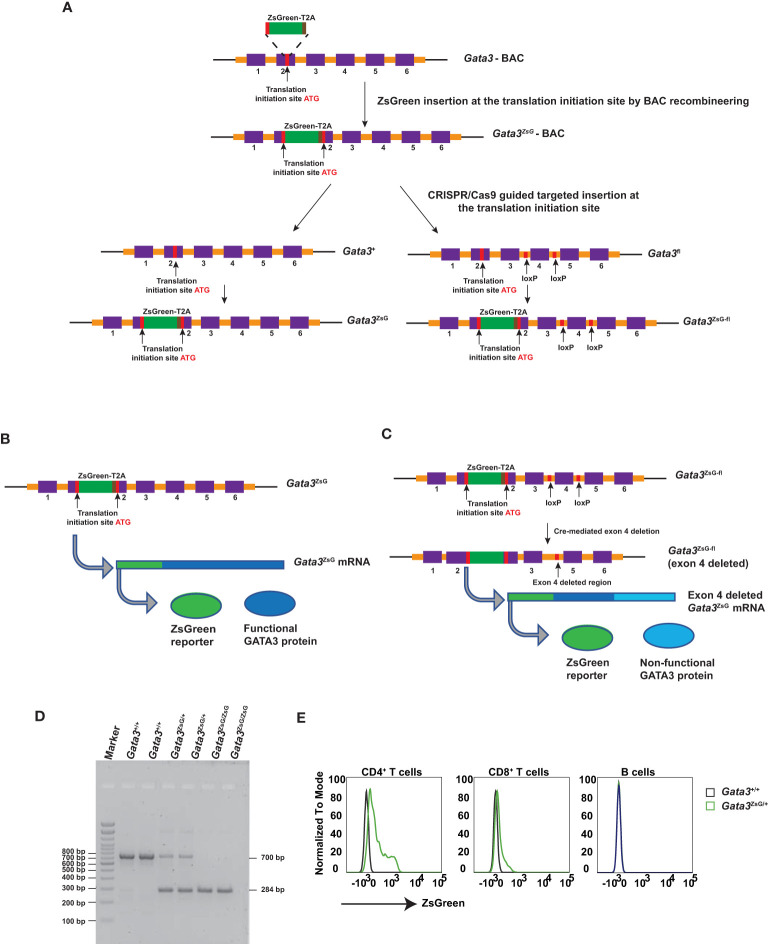
Generation of *Gata3*-*ZsGreen* reporter system on WT (*Gata3* ^ZsG^) and on conditional *Gata3* KO (*Gata3* ^ZsG-fl^) background. **(A)** Schematic representation of steps involved in the generation of mouse strains carrying the *Gata3* ^ZsG^ or *Gata3* ^ZsG-fl^ allele from the initial *Gata3* ^ZsG^-BAC construction to CRISPR/Cas9 targeted integration of ZsGreen-T2A reporter into the mouse genome. **(B)** Expected expression of ZsGreen reporter and functional GATA3 protein from the same mRNA. **(C)** Expected expression of ZsGreen reporter and non-functional GATA3 protein after exon 4 deletion. **(D)** Gel image of genotyping results distinguishing the *Gata3* ^ZsG^ reporter allele from the WT *Gata3* ^+^ allele. **(E)** Flow cytometry analysis of ZsGreen expression among CD4^+^ T cells, CD8^+^ T cells, and B lymphocytes in the blood. The results **(D, E)** are representative of more than two independent experiments.

To generate a *Gata3*-*ZsGreen* reporter system in mice, we first inserted the ZsGreen-T2A cassette into a *Gata3*-containing bacterial artificial chromosome (BAC) at translation initiation site of the *Gata3* gene (**Figure 1A**). T2A is a small self-cleavable peptide which allows co-expression of multiple proteins in a relatively equal ratio from the same messenger RNA (23, 24). Thus, the insertion of ZsGreen-T2A cassette at the ATG site of the endogenous GATA3 should result in the expression of separated ZsGreen and GATA3 protein and thus have a minimal effect on endogenous GATA3 functions. By using the CRISPR/Cas9 technology with a guided RNA targeting DNA fragment near the ATG site and the engineered BAC as the repairing template, the ZsGreen-T2A cassette was successfully inserted into the exon 2 region of either WT *Gata3* allele or the *Gata3* ^fl^ allele with conditional knockout potential (**Figure 1A**). The resulting *Gata3*-ZsGreen (*Gata3* ^ZsG^) reporter system should allow normal expression of the endogenous GATA3 protein (**Figure 1B**). On the other hand, the functional GATA3 protein on the *Gata3* ^ZsG-fl^ reporter system could be converted into a non-functional form of GATA3 after Cre-mediated excision of the exon 4 since this exon encodes zinc fingers that are critical for GATA3 functions. Because the ZsGeen is at the 5’ of the *Gata3* gene, its expression could be retained after *Gata3* deletion if functional GATA3 has a minimal role on its own transcription (**Figure 1C**).

*Gata3* ^ZsG/+^ hemizygous mice were bred with WT C57BL/6 to maintain a hemizygous state. Some hemizygous mice were also intercrossed to generate homozygous animals. The WT and ZsG alleles can be distinguished by PCR genotyping (**Figure 1D**). Since our initial testing indicated that the expression of ZsGreen from a single allele was bright enough, all the experiments in this study were carried out with the hemizygous *Gata3* ^ZsG/+^ or *Gata3* ^ZsG-fl/fl^ mice. In naïve hemizygous *Gata3* ^ZsG/+^ mice, green fluorescence was readily detectable on blood CD4^+^ T cell population by flow cytometry analysis (**Figure 1E**). Low levels of ZsGreen were also observed in CD8 T cells. However, B lymphocytes did not show any green fluorescence. These results were expected based on our previous knowledge on the differential GATA3 expression in mature lymphocytes (25–27). Furthermore, ZsGreen expression was not detected in myeloid cell populations, including neutrophils, eosinophils, DCs, and alveolar macrophages (**Supplementary Figure 1**). Therefore, these results confirm that the ZsGreen expression in our system is specific to T lymphocytes in which endogenous GATA3 is supposed to be expressed. Similar ZsGreen expression was also noted in the hemizygous *Gata3* ^ZsG-fl/fl^ mice, which were mainly used in the follow-up in-depth analysis.

### ZsGreen faithfully reflects GATA3 protein expression during thymic development

**Figure 2:**
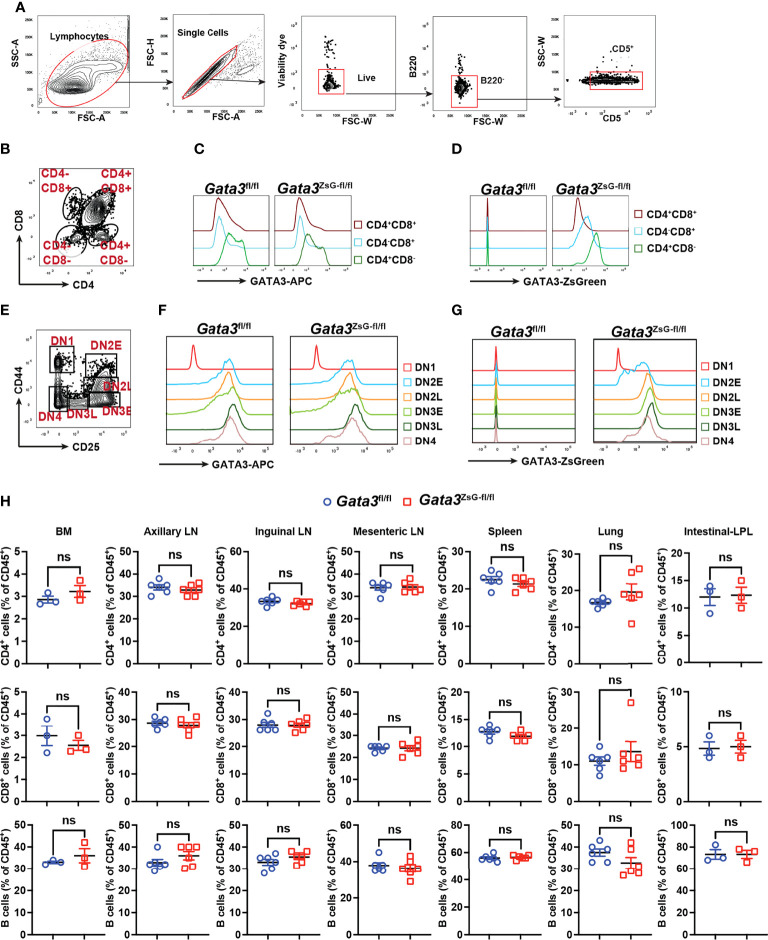
Normal thymic development in the *Gata3* ^ZsG-fl/fl^ mice. Thymocytes from *Gata3* ^fl/fl^ and *Gata3* ^ZsG-fl/fl^ mice were stained and analyzed by flow cytometry. **(A)** Gating strategy for distinguishing T cells from other cell population. **(B)** Gating strategy for analyzing different developmental stages of T lymphocytes. **(C)** Flow cytometric histograms showing GATA3 protein expression in CD4 and CD8 single or double positive lymphocytes. **(D)** ZsGreen expression in CD4 and CD8 single or double positive lymphocytes. **(E)** Gating strategy for analyzing different stages of CD4 and CD8 double negative (DN) cells. **(F)** Flow cytometric histograms showing GATA3 protein expression in DN cells at various developmental stages. **(G)** ZsGreen expression in DN cells at various developmental stages. **(H)** Distribution analysis of CD4^+^ T cells, CD8^+^ T cells, and B cells in various lymphoid organs and tissues based on flow cytometry data (Mean ± SEM; ns, not significant; Student t-test) . Results are representative of two independent experiments.

The expression of GATA3 protein is critical for the earliest T cell progenitor development, and its deficiency in hematopoietic stem cells leads to a complete absence of T cells (28, 29). The hemizygous *Gata3* ^ZsG-fl/fl^ mice had a normal level of GATA3 protein expression across various stages of thymocyte development (**Figures 2A-G** and **Supplementary Figures 2A, B**). Importantly, the ZsGreen reporter expression correlated well with dynamic changes in GATA3 protein levels during CD4 and CD8 lineage commitment (**Figures 2C, D**). Furthermore, ZsGreen also correlated well with GATA3 protein levels at different stages of CD4/CD8 double negative (DN) cells during early thymic development (**Figures 2F, G**). These results indicate that the *Gata3* ^ZsG-fl/fl^ mice have normal thymic development, and that the ZsGreen reporter faithfully reflects the expression of endogenous GATA3 throughout different stages of T cell development in the thymus. We next analyzed the distribution of T and B cells in secondary lymphoid organs and peripheral tissues. Consistent with normal T cell development in the thymus of the *Gata3* ^ZsG-fl/fl^ mice, these mice had normal numbers of B cells, CD4 T cells, and CD8 T cells in axillary lymph nodes, inguinal lymph nodes, mesenteric lymph nodes, and spleen (**Figure 2H**). Normal distribution of these lymphocytes was also confirmed in the bone marrow, lung, and intestine lamina propria.

### *Gata3* ^ZsG-fl/fl^ mice have intact ILC2 and Th2 cell response

**Figure 3:**
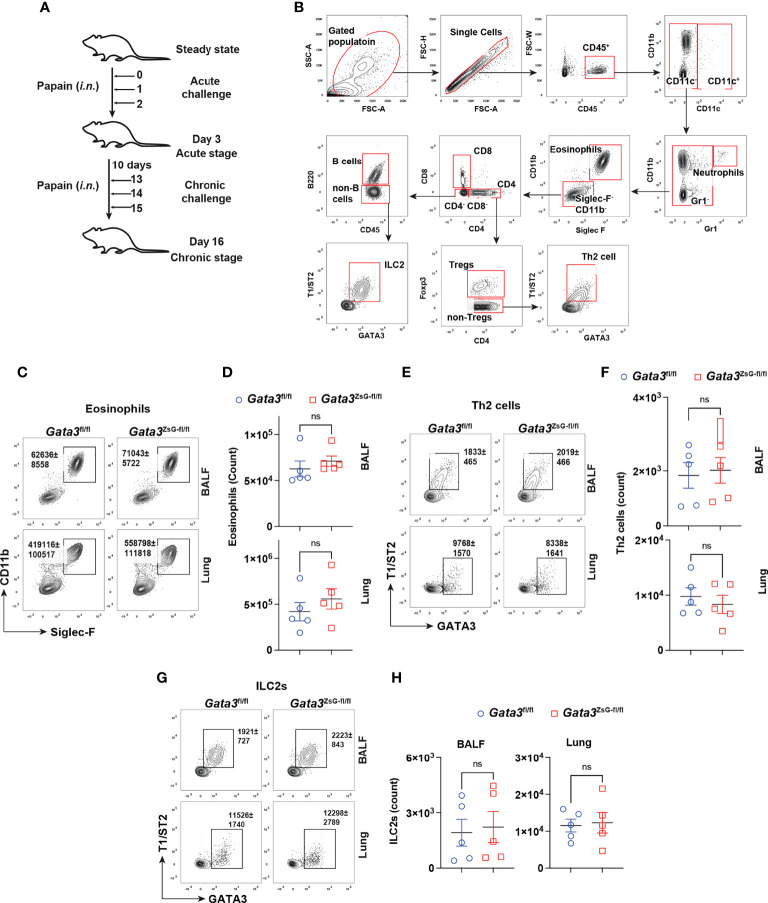
Normal development of ILC2s and Th2 cells in the *Gata3* ^ZsG-fl/fl^ mice. **(A)** Schematic representation of the mouse model of airway inflammation induced by chronic papain *i.n.* challenges. **(B)** Gating strategy for analyzing eosinophils, Th2 cells, and ILC2s from BALF and lung. **(C)** *Gata3* ^fl/fl^ and *Gata3* ^ZsG-fl/fl^ mice were challenged with papain followed by flow cytometric analysis of eosinophilic infiltration in BALF and lung. **(D)** The total numbers of eosinophils in lung in C were counted and plotted (Mean ± SEM; n = 5; ns = not significant, Student t-test). **(E)** Th2 cells in BALF and the lung were identified by flow cytometry. **(F)** The total numbers of Th2 cells in E were counted and plotted (Mean ± SEM; n = 5; ns, not significant, Student t-test). **(G)** ILC2s in BALF and the lung were identified by flow cytometry. **(H)** The total ILC2s in G were counted and plotted (Mean ± SEM; n = 5; ns, not significant, Student t-test). Results are representative of two independent experiments.

A coordinated activation of ILC2s and Th2 cells is important for generating robust type 2 immune responses (7). It has been observed that mice deficient in either ILC2s or Th2 cells show impaired immune responses (30, 31). We used a mouse model of airway inflammation by protease papain intranasal challenges, which activate both ILC2s and Th2 cells (**Figures 3A, B**). The disease severity was assessed by eosinophils enumeration (**Figures 3B-D**). Our results showed that *Gata3* ^fl/fl^ and *Gata3* ^ZsG-fl/fl^ mice had comparable numbers of eosinophils. In addition, both *Gata3* ^fl/fl^ and *Gata3* ^ZsG-fl/fl^ mice had identical numbers of ILC2s and Th2 cells (**Figures 3E-H**). Therefore, these results indicate that the insertion of ZsGreen reporter in the *Gata3* ^ZsG-fl/fl^ mice does not affect the generation of ILC2s and Th2 cells that are capable of inducing eosinophil recruitment.

### The ZsGreen reporter expression faithfully reflects the GATA3 protein levels *in vitro*

**Figure 4:**
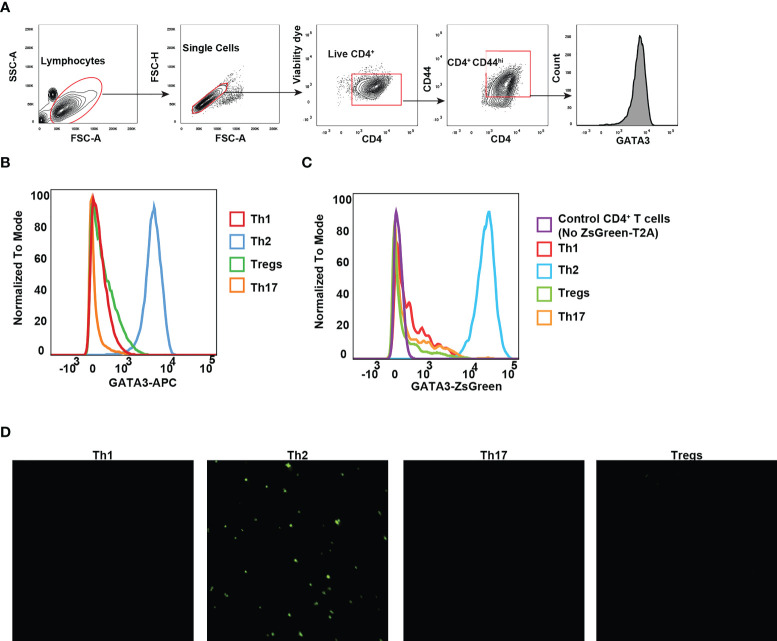
GATA3 reporter expression reflects the GATA3 protein levels. Naïve CD4 T cells from *Gata3* ^ZsG-fl/fl^ mice were polarized into different Th subsets *in vitro*. **(A)** Gating strategy for identifying live CD4^+^CD44^hi^ effector population. **(B)** Flow cytometry histograms showing GATA3 protein expression by transcription factor staining. **(C)** Flow cytometry data showing the ZsGreen reporter expression on live CD4 T cell subsets. **(D)** Representative fluorescent microscopy photographs showing the presence of green fluorescence emitting cells with Th2 polarizing conditions. Results are representative of three **(B, C)** and two **(D)** independent experiments.

The differentiation of CD4 T helper cell subsets is tightly regulated by lineage specific expression of master transcription factors: T-bet for Th1, GATA3 for Th2, RORγt for Th17, and Foxp3 for Tregs (32). GATA3 expression in CD4 T cells is induced by TCR stimulation together with IL-4 signaling, and the induction of GATA3 is critical for effector Th2 differentiation (33). To evaluate whether ZsGreen expression can faithfully reflect the GATA3 protein levels, we chose to monitor ZsGreen expression across various effector T cell populations differentiated *in vitro*, particularly because *in vitro* generated Th2 cells express high levels of GATA3. Naïve CD4 T cells from *Gata3* ^ZsG-fl/fl^ mice were isolated and cultured under different polarization conditions towards Th1, Th2, Th17, and Treg cells. As expected, Th2 cells expressed high levels of GATA3 protein (**Figures 4A, B**). Analysis of Th subsets for green fluorescence also confirmed the highest levels of ZsGreen expression in effector Th2 cells indicating that the ZsGreen reporter expression correlates with the GATA3 protein levels (**Figure 4C**). The green fluorescence expression was also preferentially detected in Th2 cells by fluorescence microscopic imaging (**Figure 4D**). These results demonstrate that the ZsGreen reporter expression in Th2 cells derived from the *Gata3* ^ZsG-fl/fl^ mice can faithfully reflect the expression of GATA3 protein.

In addition to *in vitro* differentiated T helper subsets, we also analyzed the expression of ZsGreen reporter by various ILCs in the gut lamina propria lymphocytes. The GATA3 protein judged by transcription factor staining was expressed by ILC2s at the highest levels (**Supplementary Figures 3A, B**). However, the remaining ILC subsets, including NK cells, ILC1s, ILC3s, and LTis, expressed moderate levels of GATA3 protein (**Supplementary Figures 3A, B**). Accordingly, the ZsGreen reporter expression in different ILC subsets correlated with the GATA3 protein levels with ILC2s expressing the highest level of ZsGreen among all ILC subsets (**Supplementary Figures 3C, D**). These results demonstrate that ZsGreen reporter expression from *Gata3* ^ZsG-fl/fl^ mice faithfully reflects the GATA3 protein expression in both T cell and ILC subsets.

### Functional GATA3 is dispensable for maintaining its own expression in mature ILC2s or Th2 cells

**Figure 5:**
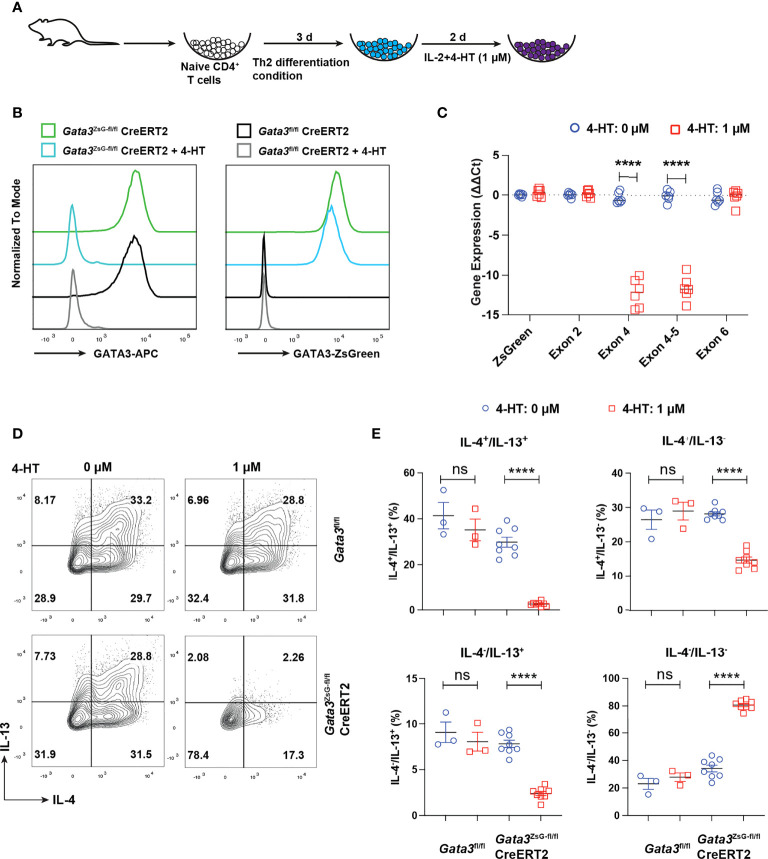
ZsGreen continues to be expressed in the GATA3 KO “Th2” cultures. **(A)** Schematic diagram for Th2 cell differentiation using naïve CD4 T cells from *Gata3* ^fl/fl^CreERT2, *Gata3* ^fl/fl^ and *Gata3* ^ZsG-fl/fl^CreERT2 mice followed by 4-HT treatment to delete *Gata3* gene in CreERT2 positive cells. **(B)** Flow cytometry histograms showing GATA3 protein levels and ZsGreen reporter expression from Th2 cells with and without 4-HT treatment. **(C)** qPCR data for analyzing the expression of ZsGreen mRNA and mRNA of indicated *Gata3* exons in the presence and absence of 4-HT (Mean ± SEM; n = 6; ****p < 0.0001, Student t-test). **(D)** Flow cytometry analysis of IL-4 and IL-13 expression by *in vitro* differentiated Th2 cells followed by 4-HT treatment. **(E)** The IL-4^+^ and IL-13^+^ Th2 cell population from **(D)** were counted and plotted (Mean ± SEM; n = 3-8; ns, not significant; ****p < 0.0001, Student t-test). Results are representative of two independent experiments.

Our previous RNA-Seq analyses of Th2 cells from WT and GATA3-deficient (deletion of *Gata3* exon 4 which encodes zinc finger domains required for GATA3 functions) mice suggest that functional GATA3 may not be required for maintaining its own transcription (2, 16, 34). To test whether ZsGreen inserted at the translation initiation site of *Gata3* can still serve as a reporter after *Gata3* gene exon 4 deletion, we isolated naïve CD4 T cells from the *Gata3* ^fl/fl^Cre-ERT2 and *Gata3* ^ZsG-fl/fl^Cre-ERT2 mice and cultured them under Th2 conditions *in vitro* for three days. The cultures were then treated with 4-hydroxy tamoxifen (4-HT) for two days (**Figure 5A**). The treatment with 4-HT, an active metabolite of tamoxifen that binds to CreERT2 to induce Cre activity, efficiently reduced the GATA3 protein in Th2 cells from both *Gata3* ^fl/fl^Cre-ERT2 and *Gata3* ^ZsG-fl/fl^Cre-ERT2 group. Nevertheless, 4-HT-treated *Gata3* ^ZsG-fl/fl^Cre-ERT2 “Th2” cells continuously expressed ZsGreen reporter after *Gata3* gene deletion (**Figure 5B**). Moreover, the mRNA quantification by RT-qPCR confirmed that 4-HT treatment effectively removed the *Gata3* exon 4. However, the expression of remaining *Gata3* exons, including exon 2 and exon 6, as well as ZsGreen reporter mRNA remained intact (**Figure 5C**). The expression of both IL-4 and IL-13 by 4-HT-treated *Gata3* ^ZsG-fl/fl^Cre-ERT2 “Th2” cells was reduced confirmed functional *Gata3* deletion (**Figures 5D, E**). We also differentiated naïve CD4 T cells from *Gata3* ^ZsG-fl/fl^Cre-ERT2 mice under Th2 conditions in the presence of 4-HT from the beginning of the culture and found that *Gata3* ^ZsG-fl/fl^Cre-ERT2 “Th2” cells had high levels of ZsGreen reporter expression even when the *Gata3* deletion occurred before Th2 cell differentiation (**Supplementary Figure 4A**). These results further demonstrate that the induction of ZsGreen expression during Th2 differentiation does not require functional GATA3.

We performed a similar experiment with ILC2s. The *Gata3* ^fl/fl^Cre-ERT2 and *Gata3* ^ZsG-fl/fl^Cre-ERT2 mice were chronically challenged with proteinase papain, and then ILC2s were purified and incubated with 4-HT for 2 days (**Supplementary Figure 5A**). 4-HT-treated ILC2s from either mouse strain no longer expressed GATA3 suggesting an efficient *Gata3* deletion, however, these ILC2s from the *Gata3* ^ZsG-fl/fl^Cre-ERT2 mice continued to express ZsGreen reporter (**Supplementary Figure 5B**). These results indicate that GATA3 is dispensable for maintaining its own expression in mature ILC2s, and that ZsGreen inserted at the 5’ of *Gata3* can be used as a valuable marker for identifying ILC2s and Th2 cells after *Gata3* deletion.

### GATA3-deficient “ILC2s” and “Th2” cells are identifiable by ZsGreen *in vivo*

**Figure 6:**
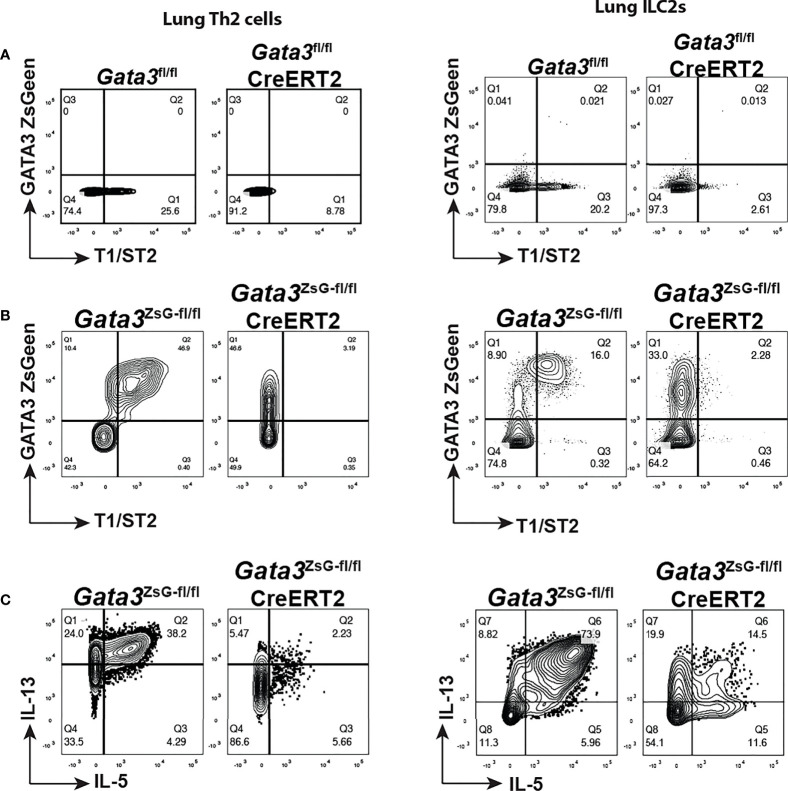
GATA3 KO “Th2” cells and “ILC2s” are identifiable in the *Gata3* ^ZsG-fl/fl^CreERT2 mice. Mice were challenged with papain as shown in **Figure 3A** and injected (*i.p.*) with tamoxifen on day 14. The mice were euthanized on day 16 to analyze ILC2 and Th2 cell responses. **(A)** *Gata3* ^fl/fl^ and *Gata3* ^fl/fl^ CreERT2 mice were challenged with papain and injected with tamoxifen followed by flow cytometric analysis of Th2 cells (left panel) and ILC2s (right panel) in lung. **(B)** *Gata3* ^ZsG-fl/fl^, and *Gata3* ^ZsG-fl/fl^ CreERT2 mice were challenged with papain and injected with tamoxifen followed by flow cytometric analysis of Th2 cells (left panel) and ILC2s (right panel) in lung. **(C)** Flow cytometric analysis of IL-5 and IL-13-expressing Th2 cells (left panel) and ILC2s (right panel) among the ZsGreen^+^ cells from the *Gata3* ^ZsG-fl/fl^ and *Gata3* ^ZsG-fl/fl^CreERT2 mice challenged with papain and injected with tamoxifen. Results are representative of two independent experiments.

To investigate the function of GATA3 in regulating its own expression in ILC2s and Th2 cells *in vivo*, we intranasally challenged mice with papain to induce airway inflammation as described in **Figure 3A** and treated mice with tamoxifen on day 14. Th2 cells induced *in vivo* in untreated mice expressed T1/ST2 as expected, and tamoxifen treatment resulted in the loss of T1/ST2-expressing CD4 T cell population (**Figure 6A**). A similar change was observed for the lung ILC2s after *Gata3* deletion. Therefore, “Th2” cells and “ILC2s” after *Gata3* deletion became unidentifiable without GATA3-ZsGreen reporter. However, in the *Gata3* ^ZsG-fl/fl^Cre-ERT2 mice while both Th2 cells and ILC2s co-expressed T1/ST2 and ZsGreen without tamoxifen treatment (**Figure 6B**), after *Gata3* deletion induced by tamoxifen, these “type 2” cells still expressed high levels of ZsGreen (albeit with a slight reduction in MFI) despite of an expected loss of T1/ST2 expression. Thus, *Gata3* ^ZsG-fl/fl^Cre-ERT2 allows us to identify “Th2” cells and “ILC2s” after *Gata3* deletion. These ZsGreen-expressing *Gata3* KO type 2 lymphocytes also had a severe reduction in the expression of type 2 effector cytokines, including IL-5 and IL-13 (**Figure 6C**). These results not only confirmed the important role of GATA3 in maintaining type 2 phenotype that was observed in our previous *in vitro* studies (2, 16, 34), they also indicate that GATA3 plays a modest role in maintaining its own expression in mature type 2 lymphocytes *in vivo*. Therefore, our novel reporter system offers a valuable tool to identify and study Th2 cells and ILC2s generated *in vivo* with and without *Gata3* deletion.

### The role of GATA3 in maintaining “ILC2” population *in vitro*

We have previously reported that GATA3 is critical for maintaining the Th2 and ILC2 population *in vitro*, but its function *in vivo* is less clear. To confirm our previous results in this new reporter system, we set up *in vitro* culture experiments by mixing the WT ILC2s from the CD45.1 congenic C57BL/6 mice and CD45.2 ILC2s from the *Gata3* ^ZsG-fl/fl^Cre-ERT2 mice in the presence and absence of 4-HT (**Supplementary Figure 6A**). Consistent with our previous report, ILC2s originated from the *Gata3* ^ZsG-fl/fl^Cre-ERT2 mice reduced dramatically in number seven days after *Gata3* deletion by 4-HT treatment as compared to the WT CD45.1 ILC2s in the same culture (**Supplementary Figures 6C, D**). Annexin V staining revealed that *Gata3* exon 4 deletion resulted in a higher frequency of apoptotic cells even two days after 4-HT treatment compared to the untreated group (**Supplementary Figures 6F, G**). We also performed mixed culture experiments with CD4 T cells differentiated under Th2 conditions and found that the persistence of Th2 cells required functional GATA3, as previously reported (**Supplementary Figure 7**). Therefore, GATA3 is indeed critical for maintaining ILC2s and Th2 cells *in vitro*.

### GATA3-deficient ZsGreen^hi^ ILCs can be maintained *in vivo*

**Figure 7:**
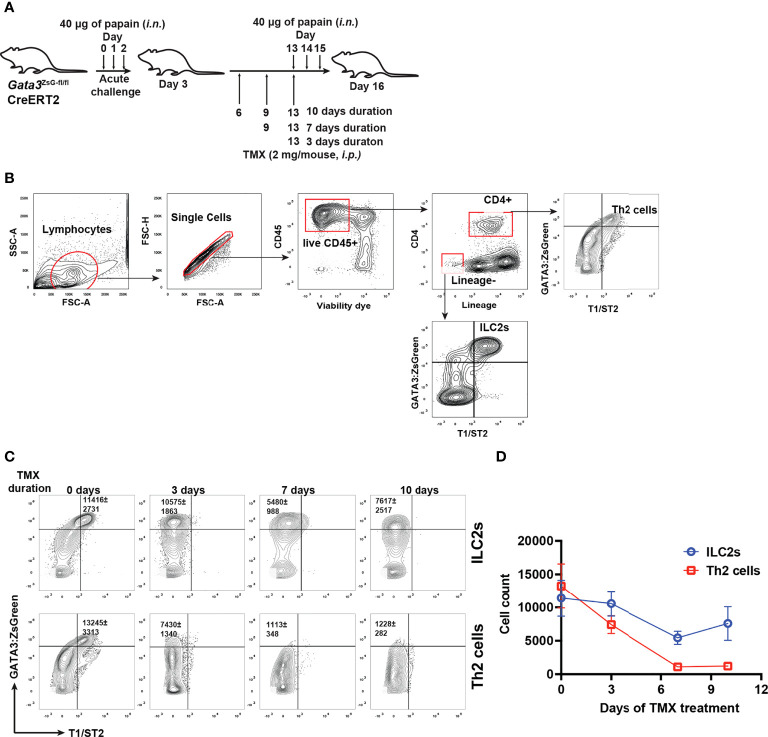
GATA3-deficient “ILC2s” are relatively stable *in vivo*. **(A)** Mice were challenged (*i.n.*) with papain and injected (*i.p.*) with tamoxifen as indicated in the figure for different durations. The mice were euthanized on day 16 to analyze the ILC2s and Th2 cells in the lung. **(B)** Gating strategy for identifying ILC2s and Th2 cells. **(C)** Flow cytometry data showing the ILC2s and Th2 cells at various time duration after TMX injection. **(D)** The total numbers of ILC2s and Th2 cells in **(C)** were counted, and the values were plotted. Results are representative of two independent experiments.

To assess whether GATA3 is also critical for maintaining ILC2s *in vivo*, we perform papain induced airway inflammation with *Gata3* ^ZsG-fl/fl^Cre-ERT2 mice that were treated with tamoxifen for various durations to assess the number of ILC2s and Th2 cells (**Figures 7A, B**). Unexpectedly, a substantial number of ZsGreen^hi^ GATA3-deficient ILCs were still identified even 7-10 days after tamoxifen treatment, although Th2 cells were barely detectable under those conditions (**Figures 7C, D**). While *Gata3* deletion in T cells early on may have blocked the generation of Th2 cells, these results also indicate that GATA3-deficient “ILC2s” might be more stable *in vivo* than *in vitro*.

### *Gata3* ^ZsG/+^ mice are also useful for visualizing ILC2s and Th2 cells by imaging

**Figure 8:**
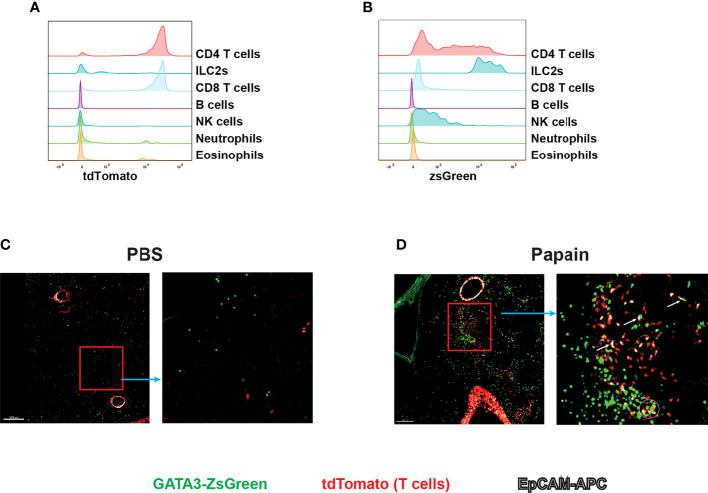
Live imaging of Th2 cells and ILC2s with *Gata3* ^ZsG/+^ *Cd4* ^Cre^ *R26* ^tdTomato^ *Gata3* ^ZsG/+^ *Cd4* ^Cre^ *R26* ^tdTomato^ mice were chronically challenges with papain and lung tissues were sliced and visualized by confocal microscopy. The green fluorescence expressing cells represents the ZsGreen-expressing ILC2s. The red fluorescence expressing cells represents the total T cells population. The colocalization of green and red fluorescent cells represent Th2 cells indicated by orange color. The white fluorescence represents the EpCAM stained cells. **(A)** Flow cytometry histogram represents the fate mapping marker tdTomato expression among various lymphoid and myeloid population in the lung tissue. **(B)** ZsGreen expression analysis by flow cytometry across various lymphoid and myeloid population. **(C)** Confocal microscope image of lung tissue slice isolated from PBS challenged mice show the distribution of ILC2s and T cell population. The highlighted part of the image was enlarged and showed beside the main image. **(D)** Confocal microscope image of lung tissue slice isolated from papain challenged mice show the distribution of ILC2s and T cell population. The highlighted part of the image was enlarged and showed on the right. Several potential interactions between ZsGreen^hi^tdTomato^neg^ cells (ILC2s) and ZsGreen^hi^tdTomato^+^ (Th2) cells were indicated by a circle and arrows in the enlarged image. Results are representative of two independent experiments.

Live imaging is an important approach for understanding the behavior and interactions of immune cells during immune responses. The reporter system allows immune cells to be observed close to physiological conditions (35). Since GATA3 is highly expressed by ILC2s and Th2 cells, our *Gata3* ^ZsG/+^ mouse strain could be a valuable tool for live imaging of ILC2s and Th2 cells. To distinguish Th2 cells from ILC2s, we have further crossed our *Gata3* ^ZsG/+^ mice to a T cell fate mapping mouse strain CD4^Cre^ *R26* ^tdTomato^. The resulting *Gata3* ^ZsG/+^ *CD4* ^Cre^ *R26* ^tdTomato^ mice allow us to distinguish GATA3^hi^ ILC2s (ZsGreen^hi^tdTomato^neg^) from Th2 cells (ZsGreen^hi^tdTomato^+^) during type 2 immune responses (**Figures 8A, B**). Indeed, papain induced dramatic expansion of ILC2s (marked by green color) in the lung of *Gata3* ^ZsG/+^ *CD4* ^Cre^ *R26* ^tdTomato^ mice, and the appearance of Th2 cells (marked by orange color, **Figures 8C, D**). Some potential interactions between ILC2s and Th2 cells were also noted (**Figure 8D**). Therefore, our *Gata3* ^ZsG^ reporter system is also suitable for live imaging of ILC2s and Th2 cells.

## Discussion

ILC2s and Th2 cells are two critical lymphocyte subsets mediating type 2 immune responses (7, 36). Both populations share the master transcription factor GATA3 for their development and functions (2–4), while it is also important for T cell and ILC development (5, 37). In this report, we prepared novel GATA3 reporter strains (*Gata3* ^ZsG/+^ and *Gata3* ^ZsG-fl/fl^). By analyzing T cell development in these reporter mice, we have verified normal expression of endogenous GATA3 protein at various stages of T cell development, which also correlated with ZsGreen reporter. Among the Th subsets polarized *in vitro*, Th2 cells expressed the highest levels of ZsGreen as expected. High levels of ZsGreen reporter expression also correlated with GATA3 protein expression *in vivo* in Th2 cells and ILC2s in papain-induced lung inflammation. Thus, these novel GATA3 reporter strains allow us to study ILC2s and Th2 cells *in vivo*.

Several cytokine reporters have been made to study Th cell differentiation, ILC development, and activation *in vivo*. However, not all the ILC or Th subsets express their signature cytokines at any given time point and such process usually requires cell activation. Therefore, transcription factor reporters are more reliable than cytokine reporters in identifying ILC and Th subsets. For lymphocytes involved in type 2 immune responses, while reporters for Th2 cytokines, including IL-4, IL-5, and IL-13, are quite valuable (38–40), GATA3 reporter should be more definitive in identifying Th2 cells and ILC2s *in vivo*.

We have previously generated T-bet and RORγt reporters by preparing BAC transgenic mice (41–43). Since these transgenic BACs contain sufficient genomic elements to mimic the endogenous T-bet or RORγt expression but are independent of natural *Tbx21* and *Rorc* gene loci, the reporters worked quite well. There is also an opportunity to breed these reporters onto the gene knockout background to study Th1 or Th17 wannabe cells. However, by using the same BAC strategy, we failed to generate a faithful GATA3 reporter strain which is consistent with the notion that the regulatory elements at the *Gata3* locus span more than 1Mb, a size that is impossible to be included in a BAC clone. Therefore, a knock-in strategy is more appropriate for preparing the GATA3 reporter. Even though we have used an engineered BAC as the repairing template in CRISPR/Cas9-mediated knock-in hoping that the very long homologous arms on the BAC can increase the efficiency of site-specific insertion, recent advances in the CRISPR/Cas9 technology allow much shorter synthetic DNAs to be used as the template.

Several GATA3 reporter strains, including *lacZ* expression system with GATA3 protein (28), GFP cassette within the *Gata3* gene locus (18), EGFP-GATA3 fusion protein expression system (17), and most recently the IRES-YFP at the 3’-UTR of *Gata3* gene locus (19), have been made. It is important to note that there is a dose effect of GATA3 in various cells. GATA3 haploinsufficiency has been reported both in mice and in humans (44, 45). Therefore, the knock-in/knock-out strategy in making a GATA3 reporter is not an ideal situation. In addition, any increased or reduced expression of endogenous GATA3 by reporter insertion could possibly have a functional impact. Both IRES and T2A have been used to introduce a reporter gene without affecting the endogenous gene expression. However, it has also been reported that the insertion of IRES sometimes may result in overexpression of the linked gene, whereas T2A has a very minimal if any effect. Therefore, we chose to use T2A over IRES in preparing our reporter mice. Nevertheless, the insertion of IRES-YFP at the 3’ of *Gata3* gene doesn’t seem to alter GATA3 expression in another report (19). Therefore, some of the previous GATA3 reporter strains have their limitations, and none of them has the potential to report GATA3 expression after conditionally deleting functional GATA3 (i.e. conditional GATA3 KO).

GATA3 regulates the expression of type 2 associated genes, including *Il5, Il13*, and *Il1rl1* in both Th2 cells and ILC2s (2). GATA3 is also critical for the expansion of Th2 cells and ILC2s *in vitro*. Such a conclusion was made based on the experiments of deleting GATA3 *in vitro*. *In vivo*, while GATA3 expression is critical for the development of ILC2s and Th2 cell differentiation, how GATA3 regulates its target genes in mature ILC2s and Th2 cells *in vivo* is difficult to address. This is partly because lack of a suitable reporter system to identify “ILC2s” and “Th2” cells after inducible *Gata3* gene deletion. It remains uncertain whether ILC2s and Th2 cells will die after GATA3 removal *in vivo*. By using the mouse strain *Gata3* ^ZsG-fl/fl^CreERT2 generated in this study, we have now shown clearly that GATA3-deficient “ILC2s” and “Th2” cells expressing ZsGreen reporter can be identified *in vivo*, and these cells indeed fail to express type 2 cytokines and T1/ST2. The continuous expression of ZsGreen after GATA3 deletion also indicates that GATA3 is not essential for regulating its own expression in mature Th2 cells and ILC2s. While ILC2s die after GATA3 removal *in vitro* consistent with previous reports, by monitoring ZsGreen^hi^ ILCs *in vivo* after GATA3 removal by tamoxifen, unexpectedly, we were still able to detect ZsGreen^hi^ ILCs ten days after tamoxifen treatment. Further investigations are needed to explain the difference between *in vitro* and *in vivo*. Can other ILCs upregulate ZsGreen expression to fill the niche if ILC2s are indeed depleted? Is the type 2 environment responsible for maintaining the survival of ZsGreen^hi^ ILCs after GATA3 removal? Are these remaining cells pre-existing ILC2s or newly generated ILC2-like cells from the ILC2 progenitors? If these remaining ZsGreen^hi^ ILCs are indeed pre-existing ILC2s before tamoxifen treatment, what are the *in vivo* signals that support their survival? It will be also interesting to test whether these “ILC2s” will gain a phenotype of other ILCs *in vivo* in the absence of GATA3.

Our new reporter strain with the conditional GATA3 knockout potential will allow the research to study GATA3-deficient ILC2s and Th2 cells *in vivo*. A similar GATA3-GFP reporter mouse strain has been previously made with the IRES-GFP cassette inserted into the 3’ of the *Gata3* exon 4, which was also flanked by two loxp sites (46). Indeed, GFP-expressing GATA3-deficient nephric duct cells can be detected in the embryos of these mice. However, it is difficult to know whether our new strain is better than this previous strain in studying lymphocytes without directly comparing them. Since the authors only reported GFP expression after GATA3 deletion, the GFP reporter is likely non-functional in the presence of floxed exon 4 given that the IRES-GFP cassette is located within the intron of endogenous *Gata3*. Therefore, when this GFP reporter is used in the GATA3-sufficient settings with a presumable *Gata3* ^gfp^/+ strain, the GFP-expressing cells from these mice are basically GATA3 heterozygous and thus express only half amounts of GATA3.

In addition, we used the ZsGreen, which is an enhanced version of GFP. Because of its brightness, ZsGreen offers an advantage in sensitivity in reporting gene expression, which also makes it suitable for live cell imaging. Indeed, after crossing the *Gata3* ^ZsG/+^ mice with the *Cd4* ^Cre^ *R26* ^tdTomato^ mice, in which all T cells are marked red, the resulting *Gata3* ^ZsG/+^ *Cd4* ^Cre^ *R26* ^tdTomato^ mice could be used to visualize ILC2s (ZsGreen^+^) and Th2 cells (ZsGreen^+^tdTomato^+^) during type 2 immune responses. Therefore, our reporter system will also facilitate future research on investigating the crosstalk between ILC2s and Th2 cells, as well as their appearance, migration, and tissue distributions under physiological conditions *in vivo*. Furthermore, because GATA3 plays an important role in early T cell and ILC development, our reporter system should also be suitable for lymphocyte progenitor studies.

## Data availability statement

The original contributions presented in the study are included in the article/**Supplementary Material**. Further inquiries can be directed to the corresponding author.

## Ethics statement

The animal study was reviewed and approved by National Institute of Allergy and Infectious Diseases (NIAID) Animal Care and Use Committee.

## Author contributions

JZ conceived the project. RG performed most of the experiments. DW and MB performed some experiments related to *in vitro* differentiation of Th subsets; QY performed some experiments related to papain-induced airway inflammation. OK performed lung tissue slicing and confocal imaging of live lung tissue. HC and MZ performed experiments analyzing ZsGreen expression by various ILCs in the gut. JKa analyzed the confocal microscopy data and produced the final images. CL and JKh supervised the generation of mouse strains carrying the *Gata3* ^ZsG-fl^ and *Gata3* ^ZsG^ allele using CRISPR/Cas9. RG and JZ wrote the manuscript. JZ supervised the project. All authors contributed to the article and approved the submitted version.

## Funding

This work is supported by the Division of Intramural Research of the NIAID (grant 1ZIA-AI-001169).

## Conflict of interest

The authors declare that the research was conducted in the absence of any commercial or financial relationships that could be construed as a potential conflict of interest.

## Publisher’s note

All claims expressed in this article are solely those of the authors and do not necessarily represent those of their affiliated organizations, or those of the publisher, the editors and the reviewers. Any product that may be evaluated in this article, or claim that may be made by its manufacturer, is not guaranteed or endorsed by the publisher.
