# Supplementary material for: Gata3 ZsG and Gata3 ZsG-fl: Novel murine Gata3 reporter alleles for identifying and studying Th2 cells and ILC2s in vivo
Source: Front Immunol. 2022 Nov 16;13:975958. doi: 10.3389/fimmu.2022.975958 (PMC9709206; doi:10.3389/fimmu.2022.975958)
Supplement: Supplementary file 1 [file Presentation_1.pdf]

Supplementory Figure 1

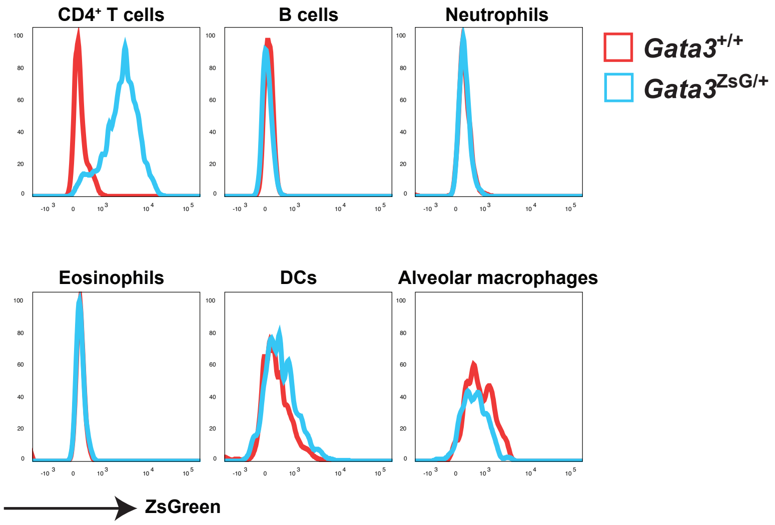

Supplementary Figure 1

ZsGreen reporter expression is specific to the GATA3-expressing cells

Flow cytometric analysis of ZsGreen reporter expression among various lymphoid and myeloid cell population.

Results are representative of two independent experiments.

Supplementory Figure 2

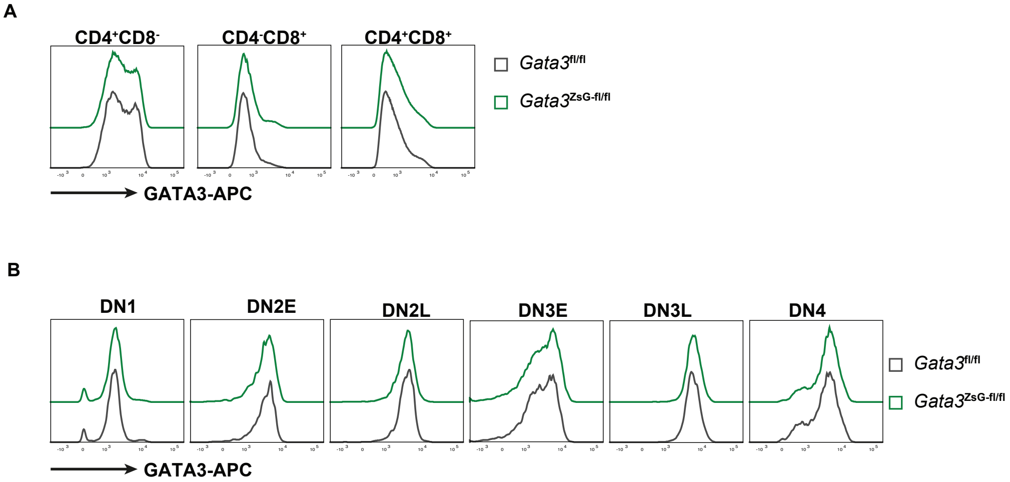

Supplementary Figure 2

ZsGreen insertion does not disrupt endogenous GATA3 expression during T cell development

A. Flow cytometric analysis of GATA3 protein expression during CD4 and CD8 committed thymocytes population.

B. Flow cytometric comparison of GATA3 levels during various stages of CD4 and CD8 double negative thymocyte population.

Results are representative of two independent experiments

Supplementory Figure 3

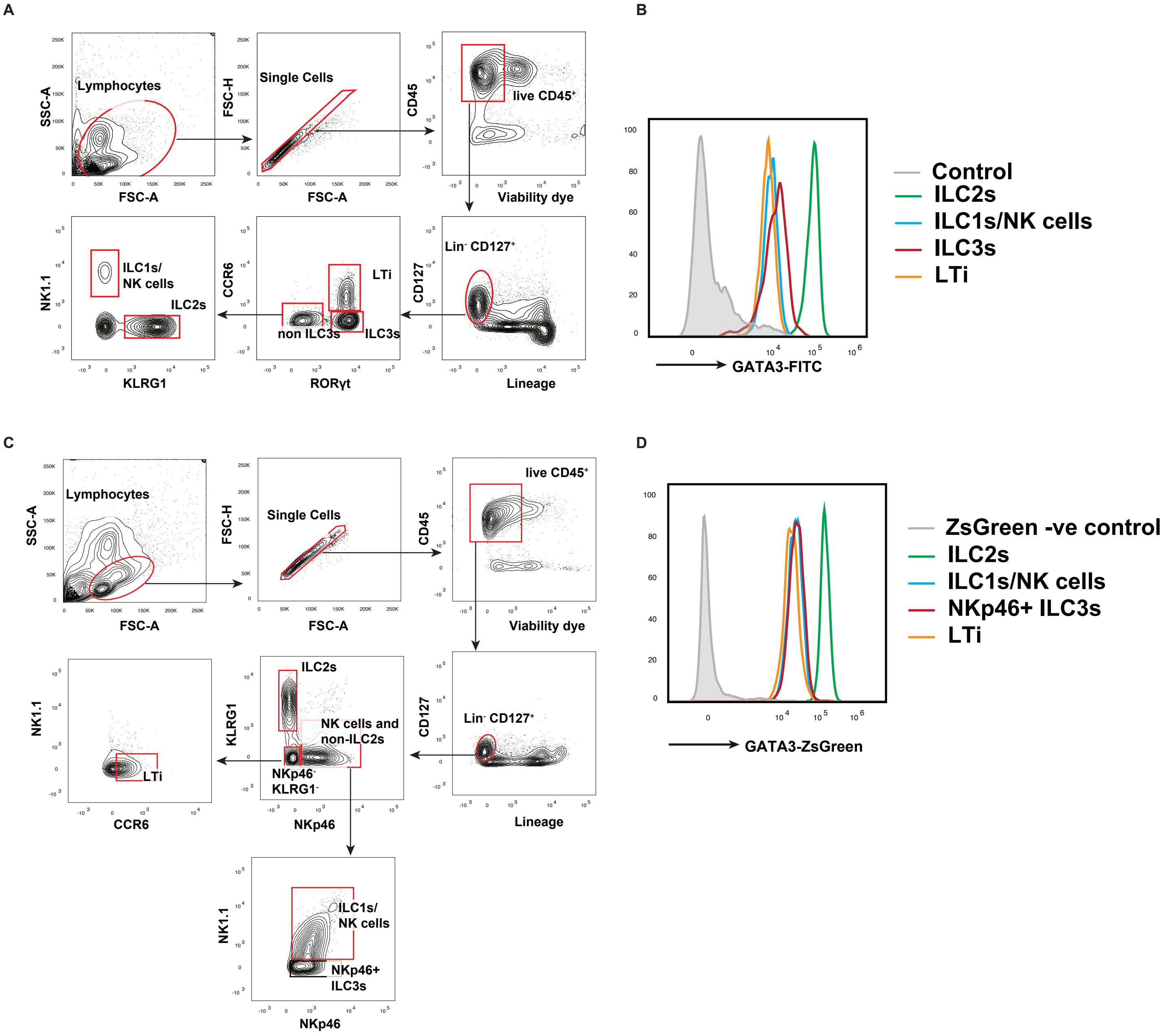

Supplementary Figure 3

ZsGreen expression by ILC subsets correlates well with GATA3 protein levels

A. Gating strategy for identifying different ILC subsets from intestinal lamina propria based on the transcription factor staining.

B. Flow cytometry histogram represents the GATA3 protein expression among various ILCs by transcription factor staining.

C. Gating strategy for identifying different ILC subsets from intestinal lamina propria based on cell surface marker staining.

D. ZsGreen expression among various ILC subsets analyzed by flow cytometry.

Results are representative of two independent experiments.

Supplementory Figure 4

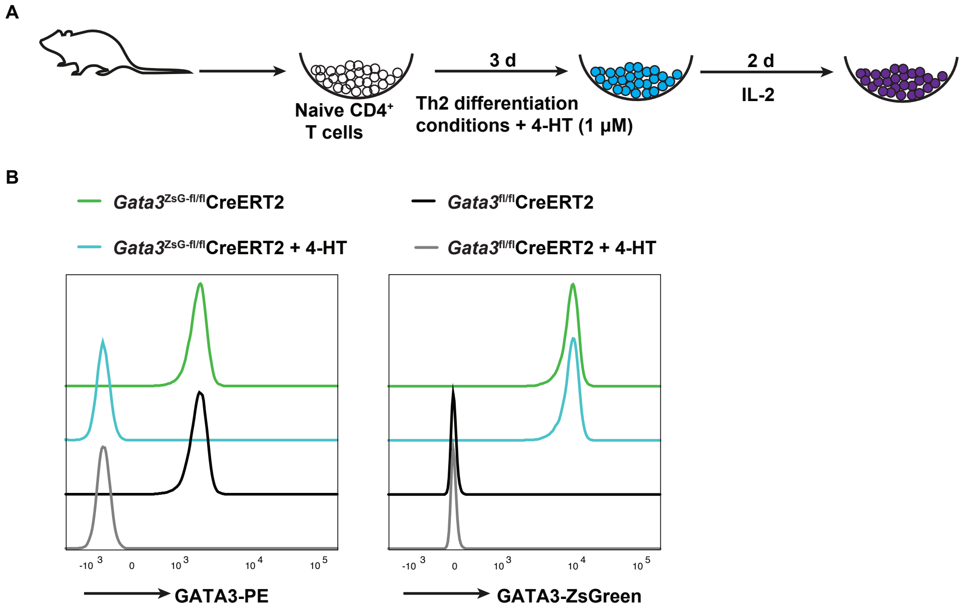

Supplementary Figure 4

Normal induction of ZsGreen expression by GATA3-deficient CD4 T cells cultured under Th2 conditions

A. Schematic diagram for naïve CD4 T cells cultured under Th2 polarized conditions in the presence of 4-HT to delete the exon 4 of the *Gata3* gene.

B. Flow cytometry histogram showing GATA3 protein levels and ZsGreen reporter expression by cells harvested at the end of culture.

Results are representative of two independent experiments.

Supplementory Figure 5

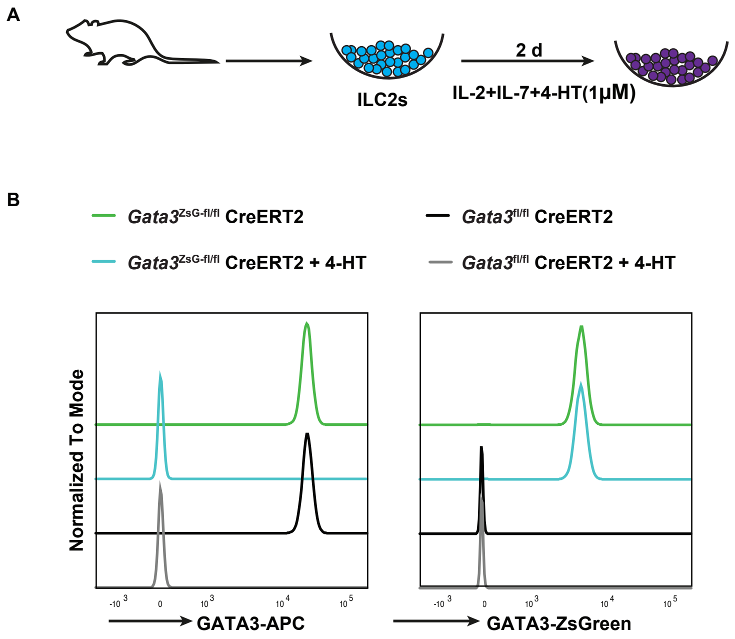

Supplementary Figure 5

ZsGreen continues to be expressed by the GATA3-deficient "ILC2s"

A. Schematic diagram for ILC2 isolation from the *Gata3*$^{fl/fl}$CreERT2 mice and *Gata3*$^{ZsG-fl/fl}$CreERT2 mice followed by 4-HT treatment to delete the exon 4 of the *Gata3* gene.

B. Flow cytometry histogram showing GATA3 protein levels and ZsGreen reporter expression from ILC2s with or without 4-HT treatment.

Results are representative of two independent experiments.

Supplementary Figure 6

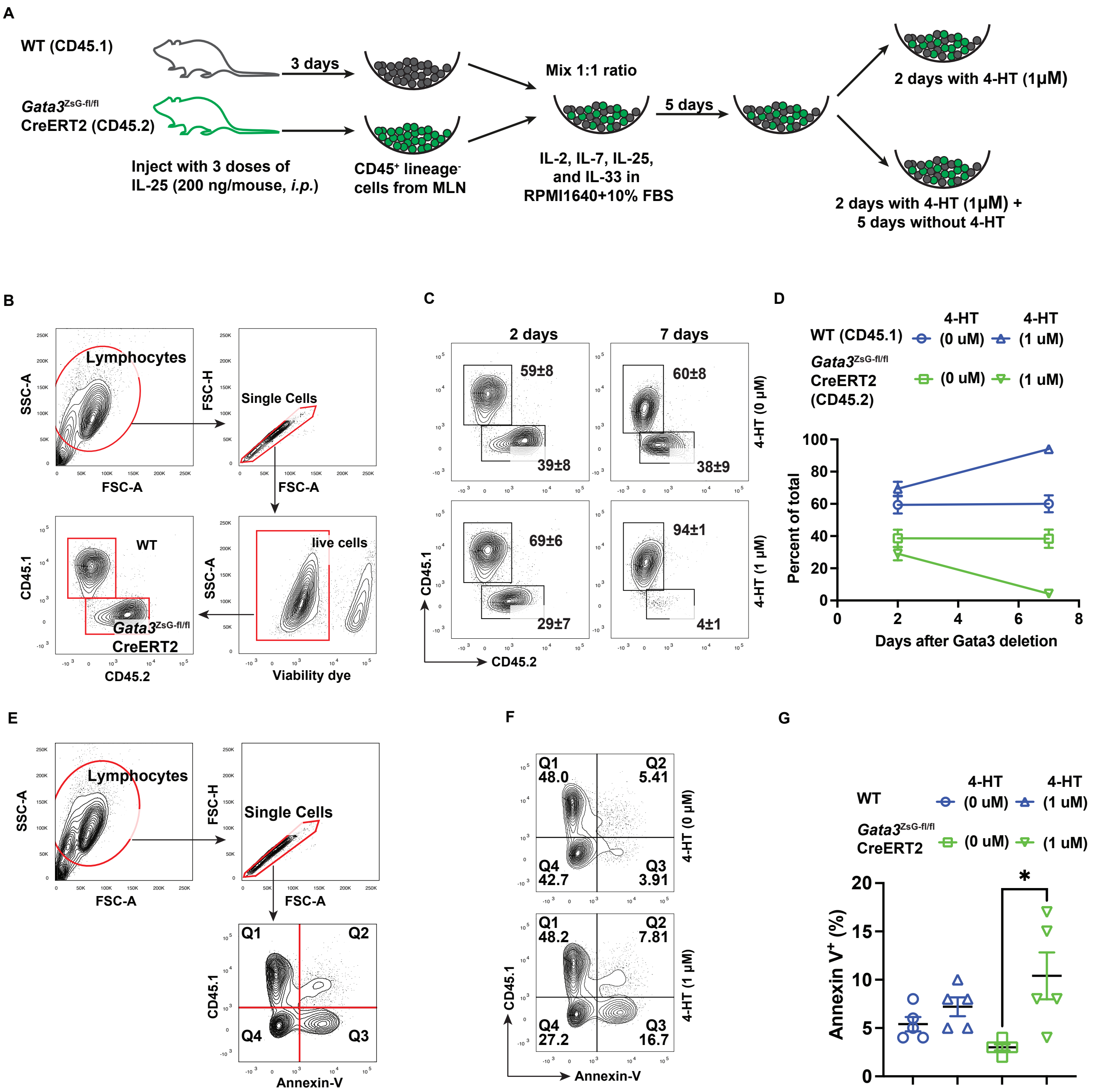

Supplementary Figure 6

GATA3-deficient "ILC2s" undergo apoptosis and decline over time *in vitro*

A. Schematic diagram represents co-culture of ILC2s isolated from IL-25-primed *Gata3*$^{ZsG-fl/fl}$CreERT2 mice (CD45.2) and WT mice (CD45.1) with and without 4-HT to delete the exon 4 of *Gata3* gene.

B. Gating strategy for distinguishing *Gata3*$^{fl/fl}$CreERT2 ILC2s (CD45.2) from WT ILC2s (CD45.1) based on the congenic marker.

C. Flow cytometry data show the relative abundance of ILC2s after *Gata3* exon 4 deletion.

D. The relative abundance of congenically marked ILC2s from C was counted, and the percentages were plotted.

E. Gating strategy for analyzing the apoptosis marker annexin V levels on cultured ILC2s.

F. Flow cytometry data for apoptosis analysis with ILC2s cultured for two days with 4-HT.

G. The percent of cells undergoing apoptosis (annexin V+) from figure F was counted, and the values were plotted.

Results are representative of two independent experiments.

Supplementary figure 7

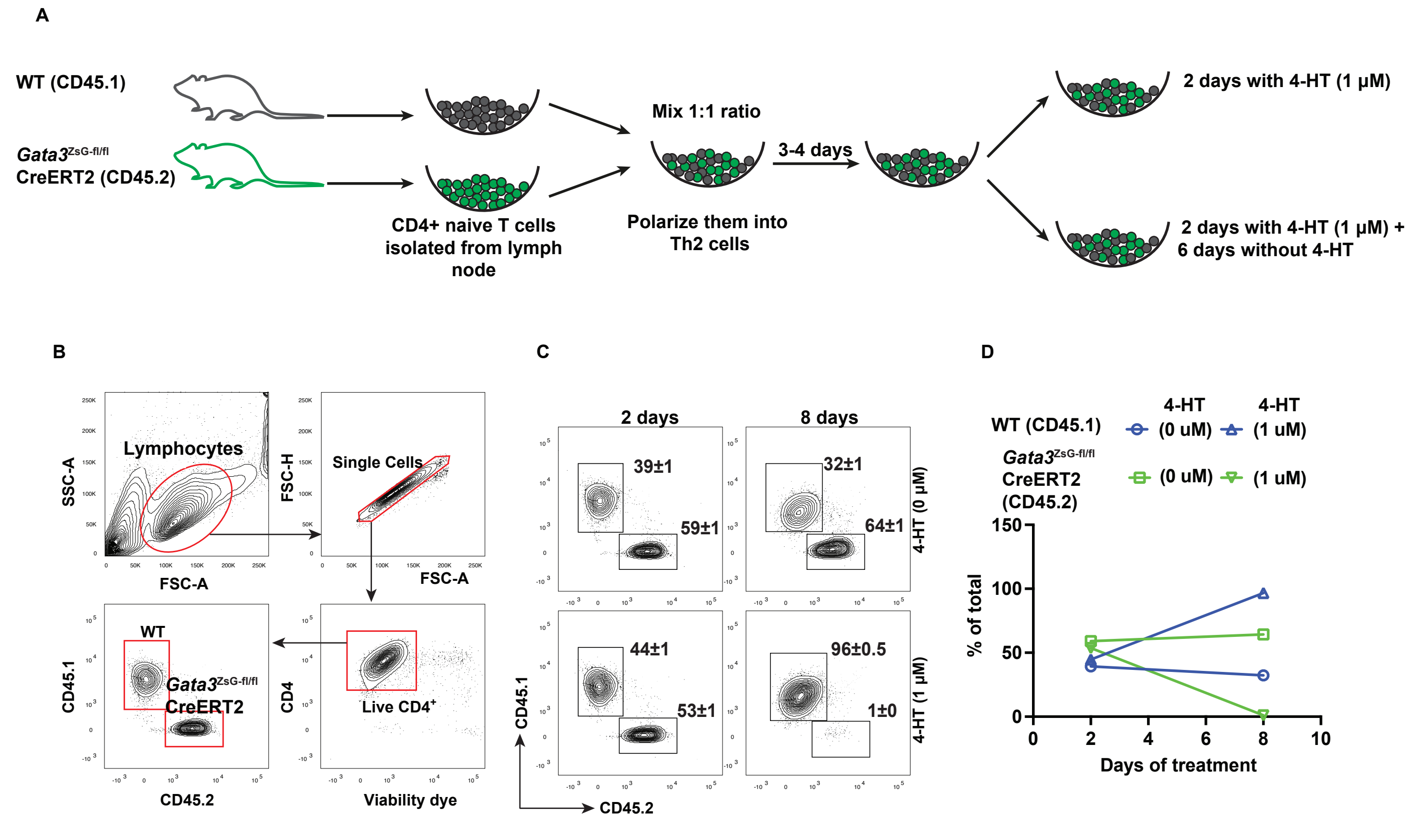

Supplementary Figure 7

GATA3-deficient "Th2" cells reduce in number over time *in vitro*

A. Schematic diagram shows the naïve CD4 T cells isolation from *Gata3*$^{fl/fl}$CreERT2 mice (CD45.2) and WT mice (CD45.1), and co-culturing them in 1:1 ratio under Th2 polarizing conditions followed by exon 4 deletion by 4-HT treatment.

B. Gating strategy for identifying the *Gata3*$^{fl/fl}$CreERT2 (CD45.2) Th2 cells from WT (CD45.1) Th2 cells.

C. The relative abundance of WT (CD45.1) and GATA3-deficient (CD45.2) Th2 cells were analyzed by flow cytometry.

D. Values from C were plotted.

Results are representative of two independent experiments.
